# Assuring assistance to healthcare and medicine: Internet of Things, Artificial Intelligence, and Artificial Intelligence of Things

**DOI:** 10.3389/frai.2024.1442254

**Published:** 2024-12-13

**Authors:** Poshan Belbase, Rajan Bhusal, Sapana Sharma Ghimire, Shreesti Sharma, Bibek Banskota

**Affiliations:** ^1^Department of Physics, Catholic University of America, Washington, DC, United States; ^2^Hospital and Rehabilitation Centre for the Disabled Children (HRDC), Banepa, Nepal; ^3^Department of Home Science, Tribhuvan University, Kathmandu, Nepal; ^4^Marie Stopes Nepal, Kathmandu, Nepal

**Keywords:** machine learning, deep learning and NLP, LLMS, AI, IOT in medicine, AIoT

## Abstract

**Introduction:**

The convergence of healthcare with the Internet of Things (IoT) and Artificial Intelligence (AI) is reshaping medical practice with promising enhanced data-driven insights, automated decision-making, and remote patient monitoring. It has the transformative potential of these technologies to revolutionize diagnosis, treatment, and patient care.

**Purpose:**

This study aims to explore the integration of IoT and AI in healthcare, outlining their applications, benefits, challenges, and potential risks. By synthesizing existing literature, this study aims to provide insights into the current landscape of AI, IoT, and AIoT in healthcare, identify areas for future research and development, and establish a framework for the effective use of AI in health.

**Method:**

A comprehensive literature review included indexed databases such as PubMed/Medline, Scopus, and Google Scholar. Key search terms related to IoT, AI, healthcare, and medicine were employed to identify relevant studies. Papers were screened based on their relevance to the specified themes, and eventually, a selected number of papers were methodically chosen for this review.

**Results:**

The integration of IoT and AI in healthcare offers significant advancements, including remote patient monitoring, personalized medicine, and operational efficiency. Wearable sensors, cloud-based data storage, and AI-driven algorithms enable real-time data collection, disease diagnosis, and treatment planning. However, challenges such as data privacy, algorithmic bias, and regulatory compliance must be addressed to ensure responsible deployment of these technologies.

**Conclusion:**

Integrating IoT and AI in healthcare holds immense promise for improving patient outcomes and optimizing healthcare delivery. Despite challenges such as data privacy concerns and algorithmic biases, the transformative potential of these technologies cannot be overstated. Clear governance frameworks, transparent AI decision-making processes, and ethical considerations are essential to mitigate risks and harness the full benefits of IoT and AI in healthcare.

## Introduction

For centuries, medicine has been a delicate dance between human intuition, expert judgment, and careful analysis. Today, a few partners have emerged on the stage: the Internet of Things (IoT), artificial intelligence (AI), and a combination of these two, artificial intelligence of things (AIoT) (Suriyan and Ramalingam, 2022). These growing technologies are redefining healthcare, promising a future where data-driven insights and automated decision-making work with human expertise to revolutionize diagnosis, treatment, and patient care (Manickam et al., 2022). While witnessing the history of healthcare systems, we can see that they face mounting pressures due to rising costs, aging populations, and a dearth of qualified professionals (Patterson, 2014; Punjani et al., 2014). Additionally, healthcare practitioners must collaborate in teams locally or distantly in the contemporary healthcare environment. This emphasizes the importance of strong communication in enabling shared decision-making, coordinated efforts, and progress evaluation. IoT helps healthcare providers manage treatments remotely, counsel patients, and monitor their conditions 24/7, making it possible to provide real-time care from a distance (Kong et al., 2022; Firouzi et al., 2023).

Traditional approaches frequently fail under the sheer volume and complexity of medical data, resulting in missed diagnoses, inadequate treatment options, and needless medical blunders (Waldram, 2000; Committee on the Learning Health Care System in America, 2013; Committee on Diagnostic Error in Health Care, 2015). In this increasingly challenging landscape, AI steps forward as a powerful tool, ready to augment human capabilities and tackle these tough hurdles (Bajwa et al., 2021). Once a futuristic dream, precision medicine becomes a tangible reality with AI algorithms that analyze mountains of genetic and clinical data, predicting how individual patients will respond to different treatments and paving the way for personalized healthcare plans (Johnson et al., 2021). Drug discovery, a notoriously long and expensive process, gets a boost from AI, with algorithms identifying promising drug candidates and optimizing their design, potentially leading to faster development of life-saving medications. Even surgery receives a futuristic upgrade with AIoT, or AI-powered robots, offering enhanced precision and control, minimizing risks, and improving outcomes for complex procedures (Blanco-González et al., 2023). Artificial intelligence, particularly large language models (LLMs), uses deep learning and neural networks trained on extensive text data from various sources to replicate human language processing. They generate coherent and realistic text by identifying patterns in the data, excelling in tasks such as machine translation and text generation in natural language processing (Kumar, 2024).

**Figure 1:**
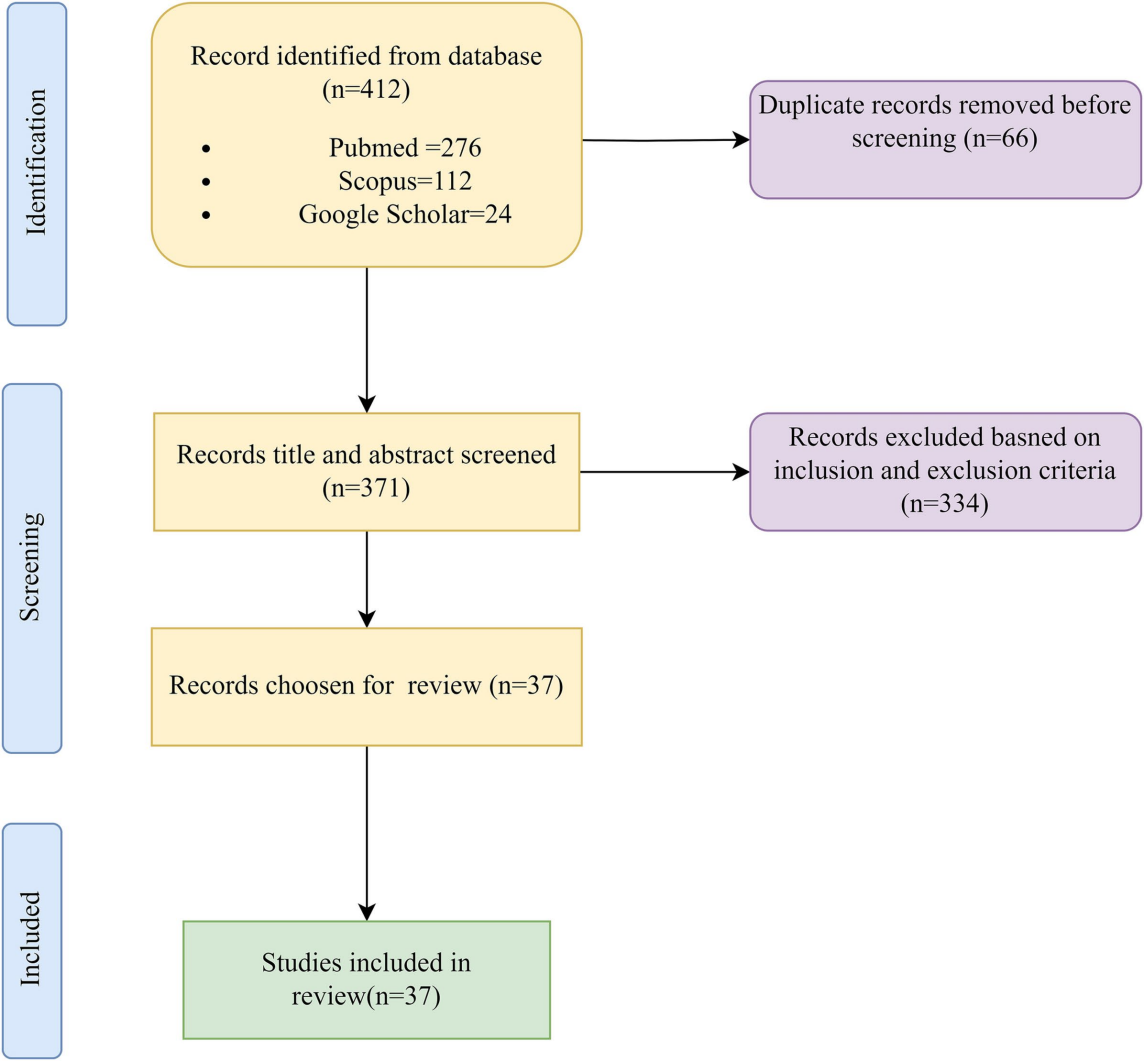
Flowchart depicting the selection process of articles for review.

On the other hand, data privacy concerns persist, ethical considerations require careful attention, and potential biases in algorithms need to be directly addressed. Robust training data and seamless integration with existing healthcare systems are essential for effective implementation (Jeyaraman et al., 2023). Building trust among healthcare professionals and patients is a non-negotiable step toward successful implementation. Despite these hurdles, the future of AI in medicine is undeniably bright. As research and development accelerate, AI will play an increasingly central role in shaping healthcare delivery (Figure 1).

### Rationale for the review

**Figure 2:**
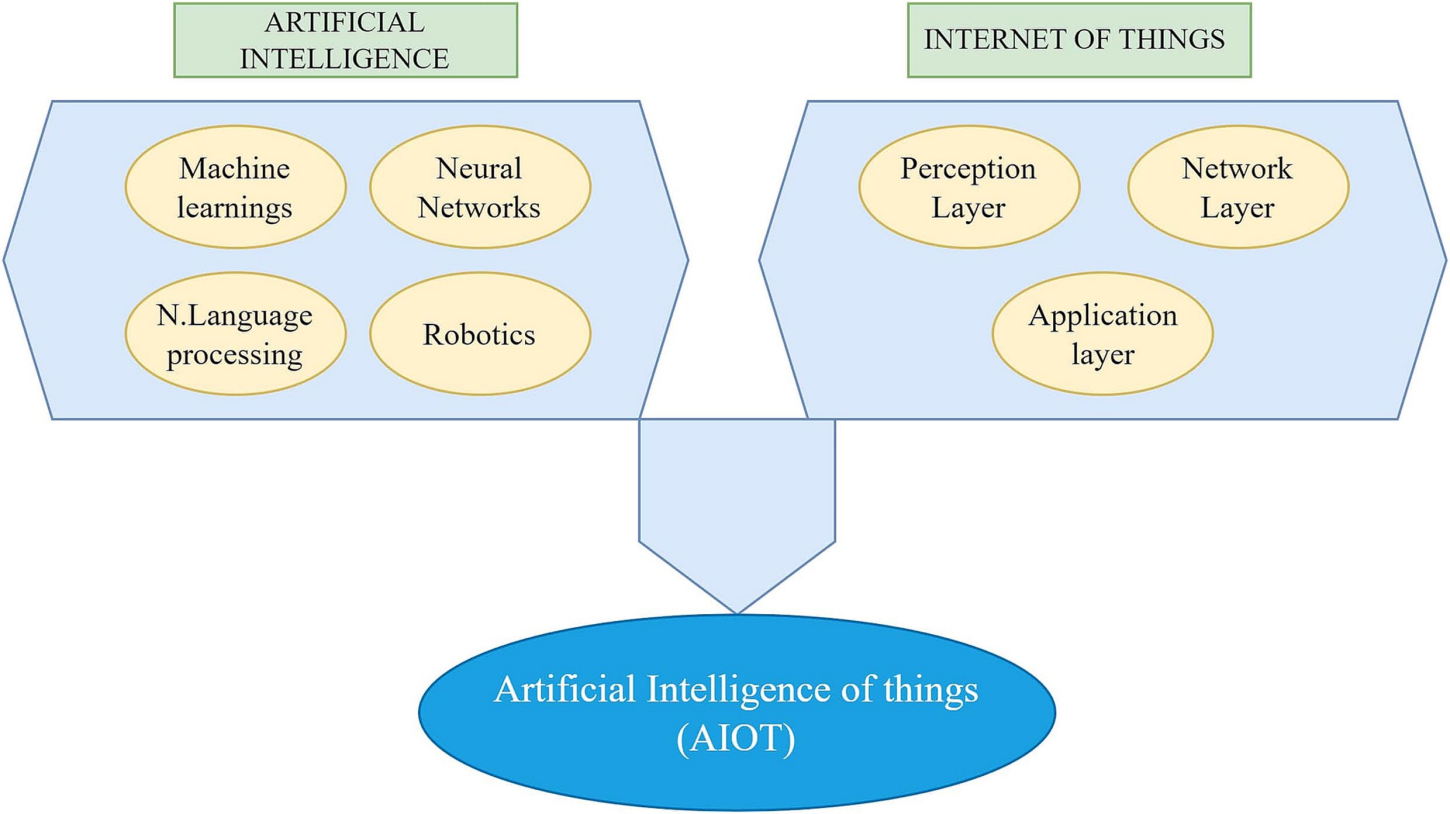
AIoT in health care and medicine.

The rapid integration of the Internet of Things (IoT), Artificial Intelligence (AI), and Artificial Intelligence of Things (AIoT) into healthcare presents immense potential to transform patient care, enhance diagnostics, and streamline healthcare delivery. However, despite the growing body of research, significant gaps warrant a comprehensive review. Existing literature lacks a structured framework for the effective and safe utilization of AI in healthcare, often failing to address the technical, ethical, and regulatory dimensions essential for its successful integration. Moreover, the careful application of AI algorithms is frequently overlooked, with insufficient emphasis on algorithmic bias, validation, and transparency, which are crucial for ensuring patient safety and equity in care. Additionally, interoperability challenges between IoT and AIoT devices and healthcare systems remain inadequately explored, hindering seamless data exchange and real-time decision-making. Furthermore, the ethical and privacy concerns surrounding the use of these technologies in sensitive healthcare settings are often underexplored, raising important questions about patient data security and consent. This review will help address these critical gaps by providing a comprehensive analysis to ensure that AI, IoT, and AIoT are deployed effectively, safely, and ethically within healthcare environments (Figure 2).

## Materials and methods

**Figure 3:**
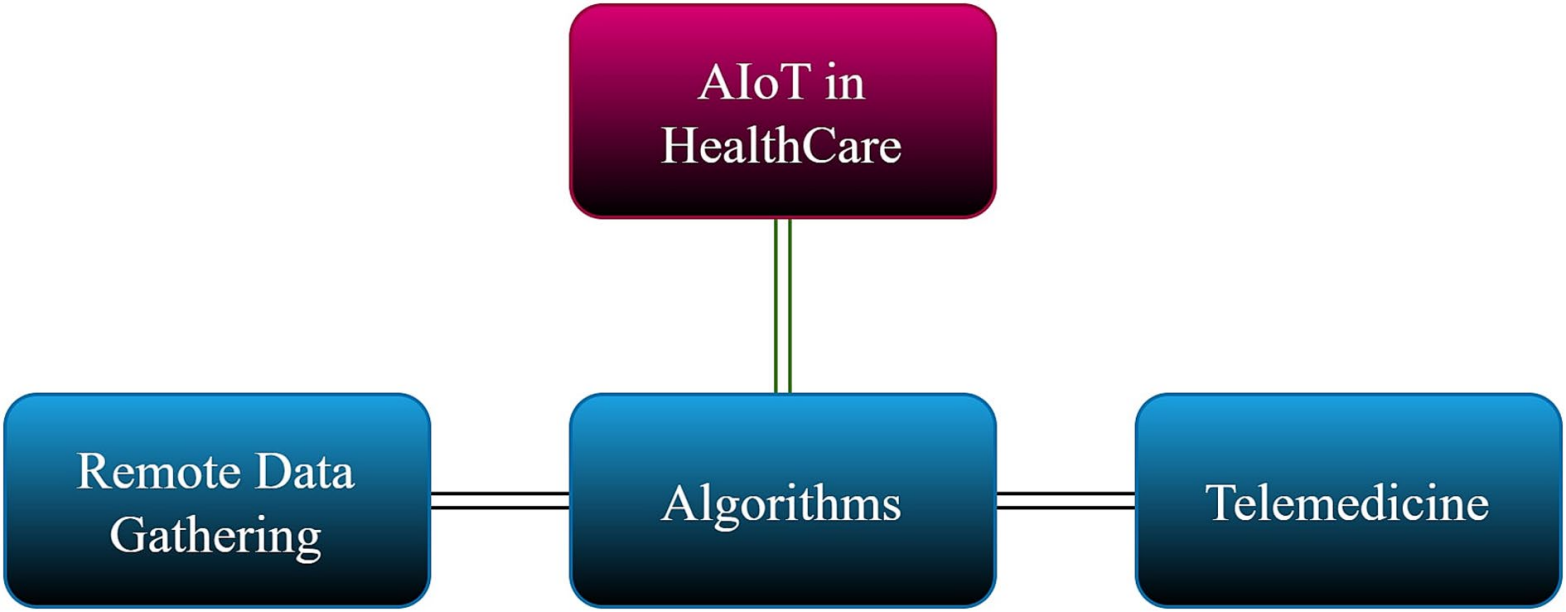
Use of AIoT in healthcare.

We identified key themes and keywords critical to shaping our research’s direction. These included technological concepts such as the Internet of Things (IoT), machine learning, deep learning, and artificial intelligence of things (AIoT), along with specific tools like natural language processing (NLP), large language models (LLMs), and ChatGPT. These terms were carefully selected to ensure the scope of our review covered the most relevant and cutting-edge advancements in healthcare technology. In addition, we prioritized research that focused on the intersection of these technologies with medicine and healthcare (Figure 3).

### Search strategy and inclusion

#### Indexed databases

Our search encompassed indexed databases such as PubMed/Medline (National Library of Medicine), Scopus, Google and Scholar. We conducted searches without temporal restrictions, though limited to English-language publications.

#### Database search protocol and keywords

The authors of this review article thoroughly explored the utilization of AI in healthcare contexts. Various combinations of keywords were employed, including IOT in healthcare, ML in healthcare, LLM in healthcare, AI in personalized medicine, AI in patient monitoring, AIoT in healthcare and medicine, AI ethics in healthcare, AI in medical diagnosis, and AI and AIoT applications in healthcare.

#### Inclusion and exclusion criteria

This review includes studies that focus on the integration of AI, IoT, AIoT, and related technologies, such as machine learning, natural language processing (NLP), and large language models, in healthcare settings. The research explored applications of these technologies in areas like personalized medicine, patient monitoring, medical diagnosis, and healthcare ethics. Eligible articles are peer-reviewed and published in journals indexed in databases such as PubMed/Medline, Scopus, and Google Scholar, with important conference papers considered due to the emerging nature of the topic. Studies must be written in English and published after 2010. Excluded from the review are publications not involving healthcare directly, such as AI applications in non-medical fields, as well as studies that lack a clear focus on the use of AI, IoT, or AIoT in healthcare, such as theoretical AI advancements without clinical relevance. Non-peer-reviewed articles, including conference papers, editorials, and commentaries, are excluded, along with papers written in languages other than English or those with insufficient methodological rigor.

#### Data extraction

Publications underwent rigorous screening, thoroughly reviewing titles and abstracts. Only those meeting specific criteria were included. Any disagreements or methodological concerns regarding the literature were thoroughly deliberated among the authors, and data was reviewed based on the themes (Table 1).

**Table 1:** Framework of using AI in healthcare.

Risk dimension	Potential risks	Mitigation measures	Expertise required	Impact level (1–5)	Likelihood level (1–5)	Risk level (Impact × likelihood)
Data privacy and security	Unauthorized access to patient health records	Implement end-to-end encryption, strict access controls, and audit trails	Data security and privacy experts			
	Data breaches leading to patient privacy violations	Regular penetration testing, continuous monitoring, and encryption of sensitive data	Cybersecurity experts, legal professionals			
	Algorithmic attacks and adversarial manipulation	Deploy adversarial training, implement model watermarking, and utilize anomaly detection.	Cybersecurity experts, AI specialists			
Accuracy and reliability	Inaccurate diagnostic predictions and treatment recommendations	Conduct extensive validation studies, use explainable AI models, and involve healthcare experts	AI specialists, healthcare professionals, domain-specific experts			
	Lack of generalization to diverse patient populations	Utilize federated learning, ensure diverse training datasets, and consider transfer learning	AI specialists, healthcare professionals			
	Model decay and drift over time	Implement continuous monitoring, automated re-training, and version control of AI models.	AI specialists, data scientists, domain-specific experts			
Ethical concerns	Unintentional bias in decision-making	Regularly audit and re-train models using debiasing techniques and enhance diversity in data.	Ethicists, AI specialists, domain-specific experts			
	Lack of transparency in AI decision processes	Develop explainable AI models, provide clear documentation, and ensure interpretability.	AI specialists, ethicists			
	Psychosocial impact on patients	Include mental health professionals in AI development teams and conduct patient feedback sessions.	Psychologists, AI specialists, healthcare professionals			
Regulatory compliance	Non-compliance with healthcare regulations	Establish a regulatory compliance team and regularly update systems to meet standards.	Regulatory experts, legal professionals			
	Changes in the regulatory landscape	Stay informed about evolving healthcare regulations and engage with regulatory bodies.	Regulatory experts, legal professionals			
Interoperability	Lack of integration with existing healthcare systems	Develop standardized APIs, follow interoperability standards, and engage with IT departments	Health informatics experts, AI specialists			
	Incompatibility with medical devices	Conduct thorough compatibility testing and adhere to medical device interoperability standards.	Biomedical engineers, AI specialists			
	System failure leading to disruption of healthcare services	Implement backup systems, disaster recovery plans, and real-time monitoring	IT professionals, AI specialists			

Scoring guideline for impact level (1-5): 1: Negligible impact 2: Minor impact 3: Moderate impact 4: Major impact 5: Catastrophic impact and for the likelihood level (1-5): 1: Rare (almost impossible) 2: Unlikely 3: Possible 4: Likely 5: Almost certain. Risk level is determined by multiplying the impact level by the likelihood level, resulting in a numerical score representing the overall risk of an event or scenario. This score varies from organization to organization based on the scale and the level of delivery. This score guides the prioritization of risks for mitigation or management efforts, ensuring urgent attention to events with high impact and likelihood.

### IoT in healthcare and medicine

The term Internet of Things was first used in 1991 by British technology pioneer Kevin Ashton and is characterized as a network of interconnected devices designed to monitor different variables (Kramp et al., 2013). IoT promises users a revolutionary, smart world with more tightly intertwined relationships between objects, their environment, and people. It can combine data from several sources, including organized and unstructured information, allowing for more consistent, streamlined, and unified access to patient data across various settings and disciplines (Coetzee and Eksteen, 2011). The rapid ascent of the IoTs can be attributed to the widespread adoption of advanced hardware and software platforms, the broadening of communication channels, and the advancements in state-of-the-art data analysis tools (Coetzee and Eksteen, 2011).

Along with multiple dimensions and fields of application, such as construction, management houses, and so on, the rise of IoT presents significant opportunities in the healthcare and medicine sector (Ray, 2018; Rejeb et al., 2022). As per the screened and selected papers, it was found that IoT mostly is wearable sensor types, chiefly accelerometer and electrocardiogram (ECG) placed on 16 different body parts, especially the wrist, chest, or implanted on the bone connected wireless with Bluetooth, cellular networks, and Wi-Fi. The messaging protocols of message queuing telemetry transport (MQTT) and constrained application protocol (CoAP) utilize the cloud to store and analyze data. The majority of the IoT have microcontrollers belonging to Atmel ATmega and ARM Cortex-M3 families; Android as the commonly used mobile operating system (OS); TinyOS and ContikiOS as the commonly used operating systems; and finally, Arduino and Raspberry Pi development boards are used trained with programming language MATLAB (Behmanesh et al., 2020). IoT is expected to play a key role in monitoring patients remotely in healthcare facilities and at home (Rose and Scott Eldridge, 2015).

As a synopsis, the implementation of IoT can give rise to various medical applications, including the management of chronic illnesses, elderly care, and fitness activities. Many patients undergoing medical treatment often need to stay in the hospital for the entire duration of their care, leading to increased hospitalization expenses. Consequently, employing technology to remotely monitor individuals with health conditions presents a potential solution. By collecting and transmitting real-time health data to clinicians, IoT has the potential to not only reduce healthcare costs but also facilitate the early detection and treatment of health issues (Dafferianto, 2014; Anurag et al., 2015).

### AI and machine learning in health care and medicine

The emulation of human intelligence in technology, such as computers or robots, is known as artificial intelligence. These devices mimic cognitive processes in human minds, like learning and problem-solving. It is common to hear AI, machine learning, and deep learning, with AI acting as a general term (Dong et al., 2020).

Algorithms created for diverse tasks such as classification, clustering, and regression are included in machine learning and require data for training. More data is provided, and machine learning algorithms perform better. Similarly, deep learning algorithms use data to train and become proficient at tasks. Artificial neural networks, like Transformers, are the foundation of advanced deep learning, a relatively new area of artificial intelligence (Wolf et al., 2020).

Over the past few years, artificial intelligence has moved from science fiction to real-world technology, a part of everyday life. This shift is not new to the healthcare industry since AI is actively used in healthcare settings. These span from automating administrative duties to supporting clinical decision-making, facilitating automated imaging, contributing to intelligent drug design, and even powering surgical robots with AI capabilities (Ahmed et al., 2020; Bhattamisra et al., 2023). Particularly in advanced economies, there is a growing interest and momentum in adopting AI technology within the healthcare sector, driven by the overarching goals of reducing costs and enhancing healthcare outcomes (Hazarika, 2020).

Supervised machine learning algorithms like Decision trees, logistic regression, K nearest neighbor (KNN), etc., used for disease diagnosis are sometimes counterparts to the capabilities of physicians in detecting cardiovascular diseases, skin cancer, breast cancer, colorectal cancer, brain cancer, cardiac arrhythmias (Lin et al., 2019). In regions with limited access to specialized care, tools embedded with these machine learning algorithms, when wielded by primary care doctors, can benefit patients significantly. University of Iowa Health Care, for instance, utilizes IDx-DR, an AI-proficient tool in detecting diabetic retinopathy, to enhance patient care. VisualDx’s Aysa app enables patients to capture images of their skin conditions, with the AI generating potential diagnoses and recommending self-care or a physician visit. Notably, Tencent’s AI can identify Parkinson’s through smartphone videos—promising advancements that can enhance care accessibility and empower primary care physicians to broaden the range of services they offer patients (Lin et al., 2019).

Using AI algorithms to analyze patient data in real-time significantly dropped emergency room (ER) visits and hospital readmission rates. Notably, the need for expensive house visits was lowered by 22%. Another notable example is Grady Hospital in Atlanta, USA, which announced $4 million in savings over two years due to a 31% decrease in readmission rates made possible by an AI-enabled application that identified “at-risk” patients (Gautam et al., 2022).

Like machine learning, deep learning techniques also play a crucial role in avoiding errors in diagnostics and enhancing test results. For example, reports indicate that deep learning techniques significantly enhance the evaluation of medical imaging, contributing to the detection of malignancy (Huang et al., 2022). Furthermore, AI enables medical professionals to manage the treatment of a higher number of patients efficiently. For instance, findings in the nursing area suggest that implementing AI-enabled solutions significantly increases productivity, ranging from 30 to 50% (Manyika, 2017). Also, artificial neural network-based voice-to-text transcription will save physicians’ and registered nurses’ labor hours by 17 and 51%, respectively (Hazarika, 2020).

Automating tedious clerical activities that sometimes overwhelm healthcare practices is one of the much-anticipated uses of AI in medicine. For example, Olive uses AI to automate several tasks, including data reporting, analytics, eligibility checks, insurance claims, prior authorizations, appointment reminders, and billing. Similarly, to help practitioners optimize their coding for quality payment programs, businesses like Apixio and 3 M have provided AI-powered solutions like risk adjustment and hierarchical condition category (HCC) auditors (Murala et al., 2023).

### AI in scientific writing and research

Artificial intelligence, especially LLMs, designed to replicate human language processing employ deep learning techniques, like neural networks, which are trained on vast text data from diverse sources such as books, articles, and websites. Through extensive training, LLMs can produce highly coherent and realistic text by analyzing patterns and connections in the data. They excel in machine translation and text generation tasks within natural language processing (Kumar, 2024).

Numerous publications have emphasized LLM-based ChatGPT’s effectiveness and potential in producing computer codes and doing thorough literature reviews, which might streamline different research procedures that usually require much human labor (Cascella et al., 2023). Furthermore, ChatGPT is an effective tool for creating accurate queries for systematic reviews, even though some writers have expressed reservations about its applicability for high-recall retrieval and transparency problems (Wang et al., 2023). AI conversational-based ChatGPT benefits nearly 85% of the records. The enhancement mainly shows up as improved scientific writing, enhanced research equity, versatility, health care research, and benefits in health care practice (Sallam, 2023). AI is transforming scientific writing and research across several domains. In data analysis and visualization, AI can process large datasets, reveal trends, and present visual insights that support hypothesis testing and data-driven decision-making. AI also streamlines literature reviews by quickly scanning and summarizing vast research, helping scholars stay current (Khalifa and Albadawy, 2024).

Moreover, AI’s ability to analyze patterns enables it to suggest new research hypotheses and optimize experimental designs, improving efficiency and minimizing bias. In manuscript writing, AI assists by generating drafts, thus speeding up the writing process, and in peer review, it can identify conflicts of interest and evaluate the quality of submissions (Khalifa and Albadawy, 2024). However, AI’s use has limitations. It lacks creativity and intuition, and its accuracy depends heavily on the quality of the data it processes. There are ethical concerns regarding AI’s role in perpetuating biases in the data. Overreliance on AI could weaken critical thinking and human judgment. AI-generated content can also be biased due to data, algorithms, and human influences (Osasona et al., 2024). To address these challenges, the scientific community must develop ethical guidelines, ensure transparency in AI systems, and foster human-AI collaboration to combine strengths. Additionally, ongoing work is needed to address bias and improve AI’s capacity to learn and adapt (Al-kfairy et al., 2024).

### AIoT in health care and medicine

AIoT (Artificial Intelligence of Things) in healthcare merges the power of artificial intelligence (AI) with the Internet of Things (IoT), creating a transformative impact on medical care. While AI primarily focuses on processing pre-collected data, AIoT collects real-time data from connected devices, such as wearables and medical sensors, and processes it using AI algorithms (Pise et al., 2022). This allows AIoT to provide real-time insights and predictions, unlike AI, which relies on historical data. In terms of personalized healthcare, AIoT enables continuous patient health monitoring, allowing treatment plans to be dynamically adjusted based on real-time data, whereas AI personalizes care based on past data. AIoT also significantly enhances remote patient monitoring by providing real-time tracking, enabling early intervention and improved outcomes.

Furthermore, AIoT shifts preventive care from identifying risks based on historical data to predicting and preventing health issues by detecting early signs and symptoms. Its applications include continuous patient monitoring, wearable health devices, AI-powered medical imaging analysis, accelerated drug discovery, and personalized treatment plans (Yelne et al., 2023). In essence, AIoT leverages the strengths of AI and IoT, allowing healthcare to become more efficient, personalized, and proactive.

Integrating IOT infrastructure and AI technologies gives rise to the concept of AIoT. An AIoT system typically consists of three main parts: algorithms, remote data collection, and telemedicine. Sensors are used in the telemedicine market to capture information or track patients’ health. In contrast, actuators are used to physically react to signals from medical professionals (in a semi-automated system) or straight from the algorithm (in a fully automated system). After pre-processing, the collected data is kept in a distributed storage system, which can be analyzed in batch or streaming mode for prediction and model construction. The gathered data forecasts each batch or cycle’s next course of action. The algorithm may be periodically re-trained using the most recent days’ worth of data or the complete dataset (Pappakrishnan et al., 2021).

In the healthcare sector, AIoT is pivotal in empowering physicians and overall hospitals while providing patients remote access to advanced scientific facilities (Lu et al., 2021). Enabling assisted living for the elderly is made possible through AIoT. IoT is a personal assistant utilizing wearable technology to monitor vital signs like heart rate, blood pressure, sleep duration, and more. It seamlessly integrates this information into a distributed data management framework, showcasing data interoperability and accessibility (Durán-Vega et al., 2019). This allows healthcare providers, researchers, and individuals to easily access and share essential health data easily, improving personalized care, enabling early detection of health issues, and promoting proactive health management.

The initial investigations into AIoT within the healthcare domain began in 2014. Subsequently, there was a notable escalation in research activities related to AIoT in healthcare during 2020–2022, a trend likely influenced by the repercussions of the COVID-19 pandemic. The uses of AIoT in pandemics and beyond have different benefits, such as early warning systems, tracking the spreading of viruses, contact tracing, screening of individuals, chatbots for diagnostics, risk prediction, bio-medical knowledge graphs, etc. (Ibrahim et al., 2022).

### Risks associated with AI and AIoT in healthcare

The potential of AI technology extends to data processing, knowledge discovery, assistive services, and the development of innovative methods to enhance healthcare quality. Traditionally, it has been the duty of certified and licensed healthcare professionals to make clinical decisions. However, as AI is increasingly incorporated into clinical activities, using AI decision support systems may impact healthcare personnel’s professional obligations to specific patients. An illustrative example is the AiCure app, developed by the National Institutes of Health, which oversees and tracks a patient’s medication usage (Mitra and Tarnach, 2022).

Despite the substantial benefits of integrating AI with medical professionals, it can occasionally yield adverse outcomes, such as IBM Watson for Oncology giving unsafe treatment recommendations and mammography AI systems having high false positive and negative rates (Zhou et al., 2019; Mayo et al., 2019). Given that AI can make incorrect conclusions, the legal responsibility for actions made with its assistance is frequently still uncertain (Koski and Murphy, 2021; BUITEN MC., 2019; Solum, 2020). The primary barrier to AI adoption is the availability and caliber of the data used to train systems. The particular characteristics of the population from which the model was created may also limit the model’s capacity to be widely applied. Clinical acceptability, explainability, validation, usefulness, ethical issues, and liability are the main issues (Hazarika, 2020; Gautam et al., 2022; Koski and Murphy, 2021). A study found that a widely used AI system for allocating healthcare resources in the US was biased against Black patients, systematically giving them lower risk scores compared to equally sick White patients. This led to unequal access to treatments and healthcare interventions (Obermeyer et al., 2019).

Along with these, many significant obstacles linked to the widespread adoption of AI and digital devices include worries about privacy, cybersecurity, data integrity, ownership, problems arising from data sharing between various organizational divisions, moral quandaries in the medical domain, responsibility for medical errors, and possible hazards stemming from system malfunctions (Gautam et al., 2022) Consumers show initial acceptance of AI as a medical provider with personalized interactions. However, neural responses reveal implicit skepticism compared to human doctors, indicating the unrealistic nature of solely replacing doctors with AI (Yun et al., 2021). Although improperly implemented AI and IoT threaten to marginalize humanity, when implemented judiciously, AI calibrates physicians’ cognitive and emotional capacities for their patients, aiding them in becoming more proficient at embodying human qualities (Lin et al., 2019; Wilson and Daugherty, 2019).

Bias in training data can lead to unequal treatment, while over-reliance on AI may undermine clinical judgment. The erosion of the patient-provider relationship is a concern, as is data fragmentation, which can lead to disjointed decisions (Ueda et al., 2024). AI’s rigidity limits its adaptability to complex cases, and ethical concerns about autonomy arise when algorithms influence treatment options. The lack of transparency in AI models hampers trust, and regulatory frameworks struggle to keep pace with AI advancements (Preprint GM, 2019). High implementation costs risk widening healthcare disparities, and AIoT systems’ reliance on stable internet and power infrastructure can lead to inconsistencies in care, particularly in underserved areas. These challenges highlight the need for responsible and equitable deployment of AI in healthcare (Khan et al., 2023).

### The framework for using AI in healthcare

This framework provides a concise roadmap for managing the risks associated with AI implementation in healthcare. It outlines specific threats, such as data privacy breaches and inaccurate diagnoses, offering tailored mitigation strategies and expertise requirements for each. By assessing risks based on impact and likelihood, the framework enables proactive risk management and ensures the development of AI systems that prioritize patient safety, ethical considerations, and regulatory compliance.

### Challenges and opportunities

#### Opportunities

The integration of AI into healthcare offers transformative opportunities, notably in enhancing diagnostic accuracy, personalizing medicine, and streamlining operational efficiencies. AI’s competence in analyzing medical imaging can lead to faster and more precise diagnoses, outpacing even expert radiologists in some instances. AIoT’s vast potential in health care and medicine includes personalized treatment plans, AI’s genetic and lifestyle data analysis, remote patient monitoring, and users in a new era of precision health care. Moreover, the use of AI in administrative and procedural tasks, such as predicting patient admissions and assisting in surgeries, will play a crucial role in optimizing resource allocation and improving patient care in the future. These advancements highlight AI’s potential to revolutionize healthcare, making it more effective, personalized, and accessible.

### Challenges

The adoption of AI in healthcare comes with significant challenges, including concerns about data privacy and security due to the reliance on extensive patient data, which risks breaches and unauthorized access. There is also the issue of bias within AI systems; if the training data is not fully representative, the AI could inherit biases, leading to inaccurate or unfair medical diagnoses. Transparency and explainability of AI decision-making processes are crucial for trust and effective use by healthcare professionals, yet many AI systems operate as “black boxes” with unclear reasoning paths. As for specificity, concerns regarding the use of ChatGPT were raised, encompassing ethical copyright, transparency, and legal issues, as well as risks related to bias, plagiarism, lack of originality, inaccurate content leading to potential hallucination, limited knowledge, incorrect citations, cybersecurity threats, and the risk of contributing to infodemics. Regulatory and governance frameworks are still evolving, necessitating clear guidelines to ensure AI’s ethical and responsible deployment in healthcare settings. These challenges underscore the complexities of integrating AI into healthcare, requiring careful navigation to harness its benefits while mitigating potential risks.

### Future research directions and potential solutions to current challenges

As the integration of the Internet of Things (IoT), Artificial Intelligence (AI), and Artificial Intelligence of Things (AIoT) in healthcare advances, addressing the inherent challenges becomes crucial for maximizing their potential benefits. Future research should enhance data privacy and security by implementing advanced encryption techniques and robust data governance frameworks to protect sensitive patient information from breaches and unauthorized access (Obermeyer et al., 2019). Additionally, efforts must be directed toward developing more representative training datasets and methodologies to reduce bias in AI systems, ensuring equitable healthcare outcomes across diverse populations (Abràmoff et al., 2023). Promoting transparency and explainability in AI decision-making processes is vital to building trust among healthcare professionals; thus, research into interpretable AI models should be prioritized (Markus et al., 2021). Addressing ethical and legal issues surrounding AI applications, particularly using tools like ChatGPT, necessitates comprehensive regulatory frameworks that provide guidelines for responsible AI deployment (Stahl and Eke, 2024). Importantly, there must not be gaps in AI integration efforts between developing and developed countries; equitable access to AI technologies and resources is essential to ensure that all populations can benefit from advancements in healthcare (Mennella et al., 2024). Overall, interdisciplinary collaborations and continuous dialog among technologists, healthcare professionals, policymakers, and ethicists are essential to navigating the complexities of AI integration in healthcare, ensuring its deployment is ethical and effective while mitigating associated risks (Korkmaz, 2024).

## Conclusion

Integrating artificial intelligence and the Internet of Things in healthcare offers significant advancements in personalized patient care, diagnostics, and operational efficiency. However, this comes with challenges such as data privacy concerns, potential biases in AI algorithms, and the need for clear regulatory frameworks. Ensuring the ethical and responsible use of AI and IoT in medicine requires a balanced approach that addresses these issues while leveraging the benefits of these technologies to improve healthcare outcomes.
